# Hepatitis B Virus Genotyping Among Chronic Hepatitis B Individuals With Resistance to Lamivudine in Shahrekord, Iran

**DOI:** 10.5812/jjm.10196

**Published:** 2014-04-01

**Authors:** Ali Karimi, Loghman Salimzadeh, Nader Bagheri

**Affiliations:** 1 Medicinal Plant Research Center, Shahrekord University of Medical Sciences, Shahrekord, IR Iran; 2 Department of Immunology, School of Public Health, Tehran University of Medical Sciences, Tehran, IR Iran

**Keywords:** Hepatitis B, Chronic, Polymerase Chain Reaction, Genotyping Techniques

## Abstract

**Background:**

Hepatitis B infection, caused by hepatitis B Virus (HBV), is one of the major global public health problems. Hepatitis B Virus genotypes appear to show varying geographic distribution with possible pathogenic and therapeutic differences. Knowledge of HBV genotypes is very important for clinical treatment. Lamivudine is a nucleoside analogue that is clinically used to treat chronic hepatitis B infection. However, the main problem with the application of lamivudine is the development of viral resistance to the treatment with this anti viral drug. Besides, it has been suggested that lamivudine -resistant HBV may be genotype dependent. However, HBV genotype distribution and the biological relevance in this region are poorly understood.

**Objectives:**

The current study aimed to determine hepatitis B genotypes and their correlation with lamivudine- resistant HBV frequency among patients with chronic hepatitis B from Shahrekord, Iran.

**Methods and Materials:**

Hepatitis B virus DNA was detected by conventional PCR in some of the serum samples obtained from HBsAg-positive Chronic Hepatitis B (CHB) patients who were referred to Health Centers of Shahrekord for routine monitoring of the disease. Subsequently, using real-time PCR, the DNA samples were used for genotyping and analysis of resistance to lamivudine.

**Results:**

The DNA was detected in 23 out of 116 (19.82%) of the studied samples. Genotypes D and C were found in 17 out of 23 (73.9%), and in 6 out of 23 (26.1%) of the samples, respectively. To the authors’ best knowledge, the current study is the first report on isolation of Genotype C from Iran. Two out of 17 (11.76%), and 6 out of 6 (100%) of genotypes D and C were resistant to lamivudine, respectively. Resistance to this drug was significantly different between genotypes C and D (P <0.001).

**Conclusions:**

In addition to genotype D, other lamivudine resistant hepatitis B genotypes might be distributed in Iran.

## 1. Background

Hepatitis B Virus (HBV) infection is a major global health problem and important infectious disease. It is estimated that there are more than 300 million people worldwide with chronic HBV infection and that 10% of these patients will die as a direct consequence of persistent viral infection (1, 2). The course of infection varies from unapparent self-limiting to chronic active hepatitis which may lead to death after many years (3, 4).

HBV is an error prone DNA virus, which undergoes genetic variability during its replication (5, 6). DNA sequencing of HBV isolates has revealed the existence of 8 viral genotypes A-H (7). HBV genotypes show varying geographic distribution (8). HBV/A is prevalent in Europe, Africa, and India (8, 9). HBV/B and HBV/C are predominant in most parts of Asia, including China and Japan (8, 10).Genotypes B and C are dominant in the Far East and south-east Asia where HBV infection is highly endemic (11, 12).Genotypes A and D are more common in Western Europe and North America. Genotype D is also predominant in the Mediterranean area as well as the Middle East, including India and Iran (13-18).

Response to antiviral treatment may be influenced by HBV genotypes (5-7). Lamivudine, a nucleoside analogue, is widely used for treatment of chronic hepatitis B patients (19-21). However, the major concern is the emergence of lamivudine-resistant HBV during the long term treatment of chronic hepatitis B (22-25). Previous studies indicated that HBV genotypes B and C influenced the response during treatment with interferon-α (26, 27). Similarly, HBV genotypes have played an important role on the response to lamivudine in the treatment of patients with chronic hepatitis B (26, 28). 

Based on HBsAg detection, Iran is located in an intermediate endemic region for chronic HBV infection in the Middle East, and patients with chronic HBV infection are presented with different clinical pictures. HBV genotyping studies on HBV-infected individuals from different regions of Iran showed that HBV genotype D is dominant in most regions of Iran (15-18). In Iran, although there are some reports on hepatitis B infection (29-31), there is no data on the distribution of HBV genotypes and genotype-dependent development of resistance to lamivudine in patients with CHB. Therefore, the present study aimed to investigate the distribution of HBV genotypes and its relation with lamivudine resistance in patients with CHB in Shahrekord. The findings of this study would provide epidemiologic data, and possibly a guideline for treatment management of the patients.

## 2. Objectives

 The current study aimed to determine hepatitis B genotypes and their correlation with lamivudine resistance frequency among patients with chronic hepatitis B from Shahrekord, Iran. The present study aimed to determine:

1. The prevalence of HBV genotypes in HBsAg-positive individuals who did not receive lamivudine treatment,
2. the rate of lamivudine resistance in these carriers, and
3. the association of HBV genotype with lamivudine resistance status.

## 3. Materials and Methods

### 3.1. Clinical Samples

A total of 116 serum samples were collected from the same number of HBsAg-positive CHB patients who were referred to the health centers of Shahrekord. Thirty six patients were female and 80 were male, with the age ranging from 25 to 71 years. Inclusion criteria were HBsAg-positive patients with anti-HCV and anti-HIV negative markers. Patients were registered irrespective of HBsAg status, ALT level, HBV DNA level or antiviral treatment status.

### 3.2. PCR Amplification and Detection of HBV DNA

HBV DNA was extracted from the serum samples using an extraction kit (QIAamp MinElute Virus Kits, Qiagen, Germany) and subjected to conventional PCR, using appropriate primers and positive control provided by the kit (plasma-serum HBV PCR detection kit, Norgen-Canada) according to the manufacturer's instructions. Strict measures were adopted to prevent any contamination. An aliquot of water was used as the negative control. Samples were considered positive if they yielded at least two positive results in two different reactions and were considered negative when there were two negative results in two different reactions. To detect the PCR product, a total of 10 microliter of the PCR products was analyzed by electrophoresis through a polyacrylamide gel, stained with silver nitrate and photographed.

### 3.3. HBV Genotyping and Detection of Lamivudine-Resistant Genotypes

Detection of HBV genotypes and lamivudine resistance was performed using quantitative real time PCR. For these purposes two commercial kits, HBV Genotype B, C and D real-time PCR kit, and HBV YMDD Mutation Real Time PCR Kit (Liferiver, Shanghai, China) were used respectively according to the manufacturer's instructions. All of the applied kits were specific ready-to-use systems that worked based on fluorogenic 5ʹ nuclease assay using specific primers and probes, provided by kits. For real-time PCR a rotor gene 3000 instrument (Corbett Research-Australia) was used. All necessary precautions to prevent cross contamination were observed; also positive and negative controls were included at each step. 

For genotyping, briefly, the kit contained two separate master mixes, one for typing of D genotype in FAM channel, and the other one for typing B in FAM channel and C in JOE channel. To detect lamivudine resistance, the kit contained single assay master mixes tube, and specific primers and probes to detect the strains with YMDD wild type motif in both FAM and JOE channels, and also the strains with YIDD or YVDD motif in only JOE channel (but negative on FAM channel). The assay results were compared with those of the kit's positive controls.

## 4. Results

**Figure 1.**
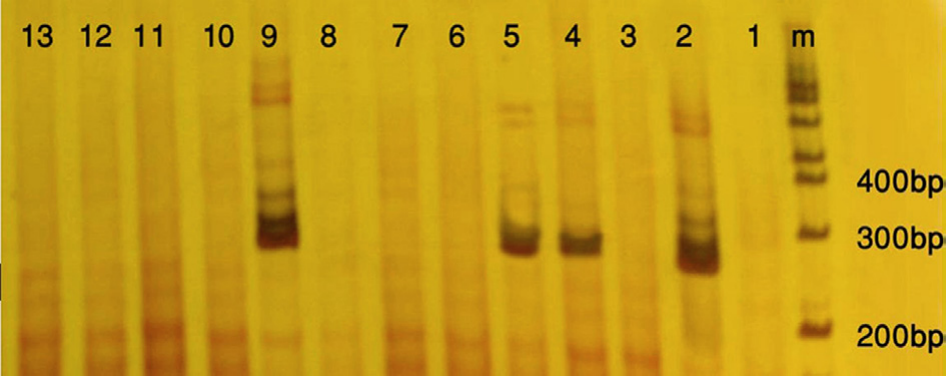
Polyacrylamide Gel Electrophoresis of Hepatitis B Virus PCR Products Line m, molecular weight DNA marker; line 1, negative control; line 2, positive control ( 285bp band); lines 4, 5 and 9, positive samples; lines 3, 6, 7, 8, 10, 11, 12 and 13, negative samples.

**Figure 2.**
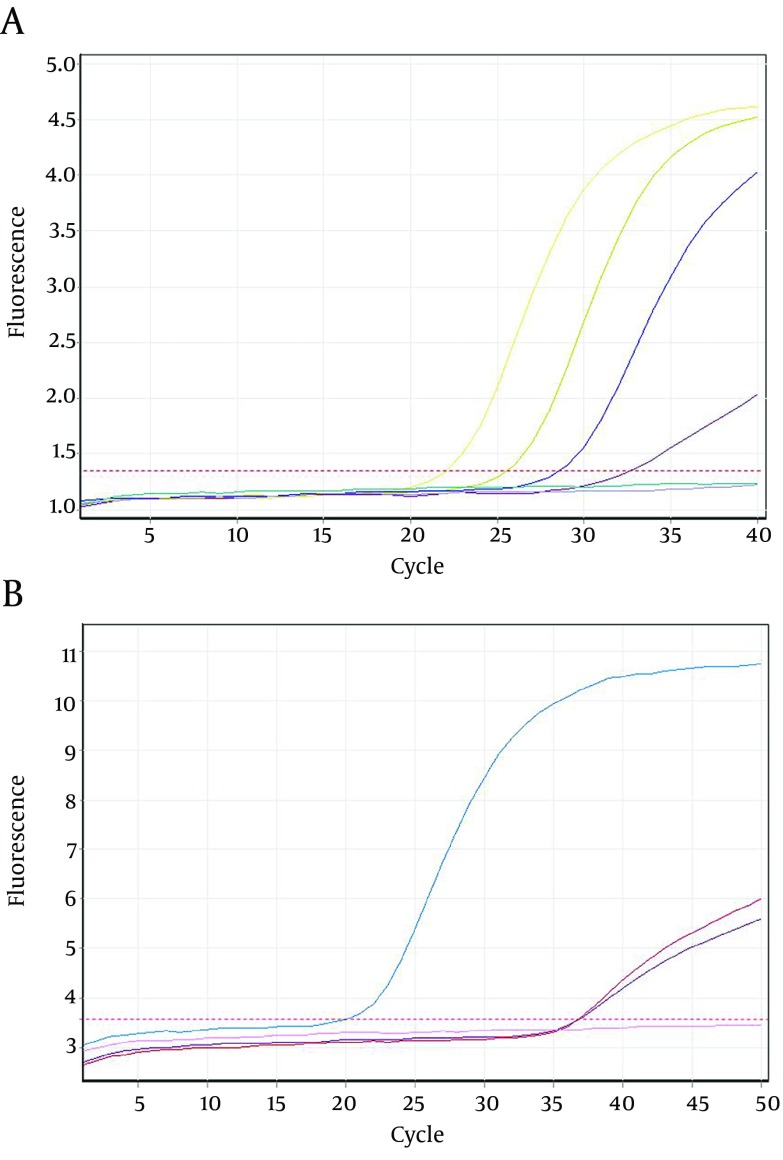
Amplification Plots of Hepatitis B Virus D and C Genotype Using Real-time PCR A. Plots were read on FAM channel. Plots upper the threshold line represent positive control and HBV strains with genotype D. B. Plots were read on JOE channel. Plots that are above the threshold line represent positive control and HBV strains with C genotype. In the both figures the plots that are under the threshold line represent negative control and samples with non-related genotypes.

**Figure 3.**
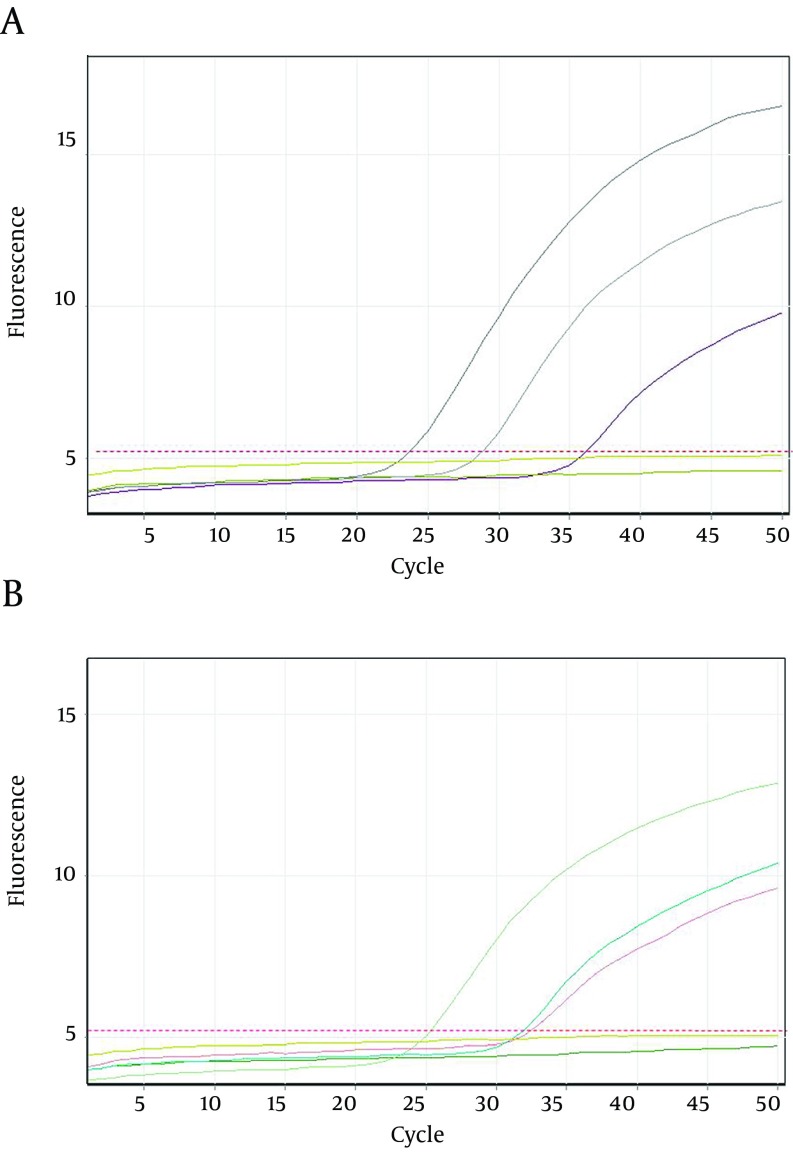
Amplification Plots of Lamivudine- Resistant Hepatitis B Virus Strains Using Real-Time PCR A. Plots were read on FAM channel. Plots represent an HBV-Y (I/V)DD mutated strain (Which is positive in FAM channel but negative in JOE channel) and two logarithmic dilutions of positive control of HBV-YMDD wild type strain (Which are positive in both FAM and JOE channels). B. Plots were read on JOE channel. The plots represent two logarithmic dilutions of positive control and one sample with YMDD wild type strain. In the both figures the plots that are under the threshold line represent negative control and samples with non-related HBV strains.

Twenty seven out of 116 (23.2%) of the HBsAg positive individuals were female and their mean age was 39.11 years. Twenty three out of 116 patients (19.82%) were positive for HBV DNA in the conventional PCR (Figure 1). Using real-time PCR, 17 out of 23 (73.9%) were infected with HBV genotype D (Figure 2) and 6 out of 23 (26.1%) were infected with HBV genotype C (Figure 3). To the authors` best knowledge, this is the first report on the isolation of genotype C in Iran. Eight out of 23 (34.7%) of HBV DNA positive samples were resistant to lamivudine. Two out of 17 (11.76%) and 6 out of 6 (100%) of genotypes D and C were resistant to lamivudine respectively. Resistance to this drug was significantly different in genotypes C and D (P < 0.001).

## 5. Discussion

Variation of HBV genome during viral replication leads to creation of different genotypes with different geographical distribution (1, 2). Clinical picture, prognosis of the disease and response to antiviral treatment may be genotype dependent (21). Accordingly, the current study investigated the distribution of HBV genotypes and their resistance to lamivudine in a group of CHB patients who did not receive lamivudine in a central province of Iran. 

 The current study results showed that genotype D is the most prevalent genotype in this region, since it was found in 73.9% of the CHB patients. Consistent with results of this report, it has been shown that this genotype is the most prevalent genotype worldwide (13, 14) and also in the most regions of Iran (15-18). However, as the current study results showed, it may not be the only genotype distributed in Iran. Based on the results of the current study, 26.1% of these patients were infected with HBV genotype C. To the authors best knowledge, this is the first report on this genotype from Iran. Genotype C is one of the most prevalent genotypes in Thailand and China (8-12). It has been also suggested that distribution of HBV genotype is influenced by immigrant population (8-10). Since a large number of Iranian have traveled to these two countries in the recent years, there is the possibility that distribution of this genotype in our region originated from these two countries.

Error-prone replication of HBV leads to appearance of lamivudine resistant strains of this virus (32, 33). Some published results indicated that lamivudine resistant mutant existed in the serum of CHB patients who did not receive lamivudine treatment (32, 34). In the present study, eight out of 23 (34.7%) of HBV DNA from CHB individuals who did not receive lamivudine, showed this mutation. Therefore, it might be concluded that naturally occurring HBV lamivudine resistant mutants are circulated in the region as well. According to the results of the current study, 11.76% of HBV genotype D and 100% of genotype C were resistant to lamivudine, indicating that lamivudine resistance was significantly different in genotypes C and D (P <0.001). There are some reports indicating that resistant to this antiviral among HBV genotypes is significantly different (24, 26, 28) and that the rate of resistance to this drug is lower in patients infected with genotype D than in patients with some other genotypes which is in agreement with those of the current study findings.

The results of the study might provide epidemiologic data on the distribution of HBV genotypes in this region. In conclusion, based on the obtained results HBV genotype D might not be the only genotype distributed in most regions of Iran, and some other genotypes such as genotype C might be present in this country. In addition, the current study findings, in accordance with many other published ones, would provide supportive evidences indicating that HBV lamivudine resistance is genotype dependent and naturally occurring HBV lamivudine resistant mutants might circulate in our country as well.
